# Faunus: An object oriented framework for molecular simulation

**DOI:** 10.1186/1751-0473-3-1

**Published:** 2008-02-01

**Authors:** Mikael Lund, Martin Trulsson, Björn Persson

**Affiliations:** 1 Institute of Organic Chemistry and Biochemistry, The Academy of Sciences of the Czech Republic, Flemingovo nam.2, CZ-16610 Prague 6, Czech Republic; 2 Department of Theoretical Chemistry, University of Lund, P.O.B 124 SE-22100 Lund, Sweden

## Abstract

**Background:**

We present a C++ class library for Monte Carlo simulation of molecular systems, including proteins in solution. The design is generic and highly modular, enabling multiple developers to easily implement additional features. The statistical mechanical methods are documented by extensive use of code comments that – subsequently – are collected to automatically build a web-based manual.

**Results:**

We show how an object oriented design can be used to create an intuitively appealing coding framework for molecular simulation. This is exemplified in a *minimalistic *C++ program that can calculate protein protonation states. We further discuss performance issues related to high level coding abstraction.

**Conclusion:**

C++ and the Standard Template Library (STL) provide a high-performance platform for generic molecular modeling. Automatic generation of code documentation from inline comments has proven particularly useful in that no separate manual needs to be maintained.

## Background

Molecular simulation has become a standard tool for investigating molecular systems such as proteins, polymer solutions and other colloidal particles. It is safe to say that for biological applications Molecular Dynamics (MD) is by far the most popular method as it provides both static and dynamic properties of the system. Metropolis Monte Carlo (MC) simulation [1], on the other hand, is less utilized and relatively few software packages exists [2-4]. One advantage of MC simulation is that it allows "unphysical" particle moves, enabling a more creative sampling of the configurational space [5]. The tradeoff for this freedom to move particles is the loss of all dynamic information and, in addition, MC programs tend to become less general. However, if one is interested in equilibrium properties only – binding constants, free energy changes, p*K*_*a *_values etc. – MC simulation may be a good option.

Using a standard, pre-compiled software package should require no prior knowledge of programming and as such can be a fast and practical approach for solving a specific scientific problem. On the other hand, the underlying physical theory is somewhat hidden and there is always a risk that the application is regarded as a "black box" producing numbers. It becomes even worse if new features are to be implemented. The alternative is for researchers to create their own programs. This approach of course requires some programming skills and writing an advanced simulation program from scratch may be an overwhelming -and likely error prone – task. Instead the programmer may resort to existing libraries, thus approaching the black box situation described above. However, the abstraction level will typically be lower which has several advantages that allows the researcher to (i) have a high level of control, and (ii) experience high performance due a minimalistic design. In this text we present a modular C++ [6] framework or class library that can be used to construct MC simulation programs in an expeditious manner. Other C/C++ libraries for molecular simulation do exist: MDAPI [7], OOMPAA [8], Glotzilla, for example, albeit none of these target Monte Carlo simulation specifically. Due to the common language, faunus can easily interweave these libraries to broaden the intrinsic feature set with additional well-proven code. A successful example of incorporating features from an external library, Gromacs GMX [9], is presented in the text.

## Implementation

### Object oriented design

The object oriented capabilities of C++ have enabled us to create a more appealing interface than traditional procedural approaches. For example, the handling of particles – a key undertaking of all classical simulations – is provided by a class hierarchy:

class point {

public:

   double x, y, z;

   double dist(point &);

   ...

};

class particle : public point {

public:

   double charge, radius;

   ...

};

The Standard Template Library (STL) is subsequently used to construct a vector of particles, **vector<particle>**, that allows for easy access and manipulation. For instance, **p [i].radius** will return the size of the *i*'th particle.

### Polymorphic classes – Virtual functions

**Figure 1:**
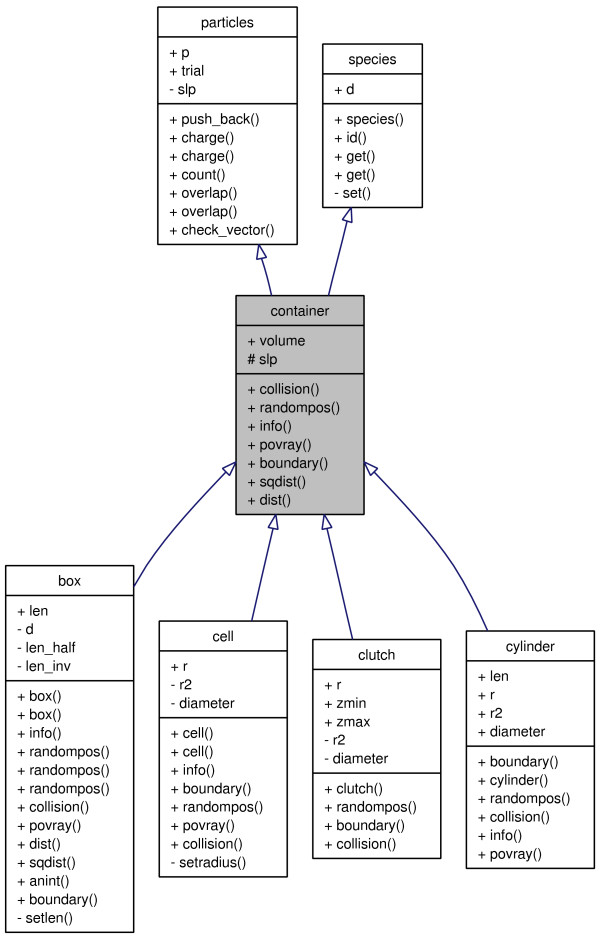
**Graphical class hierarchy**. Schematic representation of class inheritance used for the container class. Intuitive inheritance is used whenever possible. For example, a container contains particles, it can have a shape etc. The graphical representation is produced using Doxygen.

One of the unique features of C++ is *polymorph classes *that allows for very generic and intuitively appealing code. To demonstrate this, we outline the design of our framework for handling the simulation container – see Figure 1. Essentially, the end programmer will want to select among different geometries -a box, sphere, cylinder etc. For each geometry we need functions that can calculate the volume, generate a random point or decide whether a given point falls within the boundaries. We now construct a polymorph class, **container**, that defines the unimplemented *virtual functions*. Derived classes – **box, cylinder** etc. -then implement specialized versions of the functions and the **container** class hence acts as an interface to the various geometries. This means that we can construct functions that accept any geometry derived from the **container** class. For example:

double concentration(container &c)

{ return N/c.volume(); }

Due to a large overhead, virtual functions may, however, negatively impact performance and are generally avoided in critical, inner loops.

### Performance aspects

#### Function inlining via templates

The most computationally demanding step in most molecular simulations is the evaluation of configurational energies. Hence the applied pair potential must be highly optimized and preferably inlined in all inner loops. This is accomplished by passing a pair potential *class *as a template parameter that creates a local instance inside the inner loop template,

class coulomb {

    float energy(particle &a, particle &b)

    {

      return a.charge*b.charge/a.dist(b);

   }

};

template<class T_pairpot>

class innerloop {

   T_pairpot pair;

   float sum(vector<particle> &p) {

      for (i = 0; i<N-1; i++)

         for (j = i+1; j<N; j++)

            u = u+pair.energy(p [i], p [j]);

      ...

};

This so-called Expression Template technique [10] enables the programmer to arbitrarily invoke various pair potential functions, re-cycling the inner loop implementation,

innerloop<coulomb> elec;

innerloop<lennardjones> lj;

elec.sum(p);

...

#### Passing arguments as references

In C++ standard argument passing is done by creating a new copy of the object. Working with large, aggregate structures such as particle vectors, this will negatively impact performance. To circumvent this we pass all complex objects as references, for example: **void function(someclass &)**.

#### Minimize memory consumption

Computer simulations of classical mechanical systems are usually not memory intensive and by minimizing the memory requirements there is a good chance that the executing code will stay in the local cache. Code re-cycling via class enheritage is extensively utilized to reduce the memory imprint as well as assist efficient development. In this regard C++ templates are a concern since code will be generated for each template type. We therefore stride to avoid extensive use of multiple template types – for example it would seem silly to instantiate both a **float** and a **double** version of an elaborate template class.

Only for systems with tens of thousands of atoms the particle coordinate vector may extend beyond the local cache – for example, one megabyte of cache can encompass 1024^2^/8/3 ≈ 43700 double precision three dimensional particle coordinates. List methods and additional single particle information may decrease this number and, while not yet implemented in faunus, sophisticated methods do exist for cache efficient bookkeeping of many particle systems [11].

#### Parallelization

In systems that equilibrate fast, Monte Carlo simulations can be linearly parallelized using the "embarrassingly simple technique" – that is start several independent runs with different random seeds, combining the results afterwards. Tightly coupled parallelization is incorporated in parts of the code by threading the energy evaluation into two processes: before and after a trial move. For systems with particles in the order of hundreds, this scales well on dual-core computers, whereas the overhead becomes unacceptable for small systems. To enable threading, the appropriate compiler flag for OpenMP [12] must be set; as of writing the GNU, Intel and IBM C++ compilers all support OpenMP.

### Code documentation

**Figure 2:**
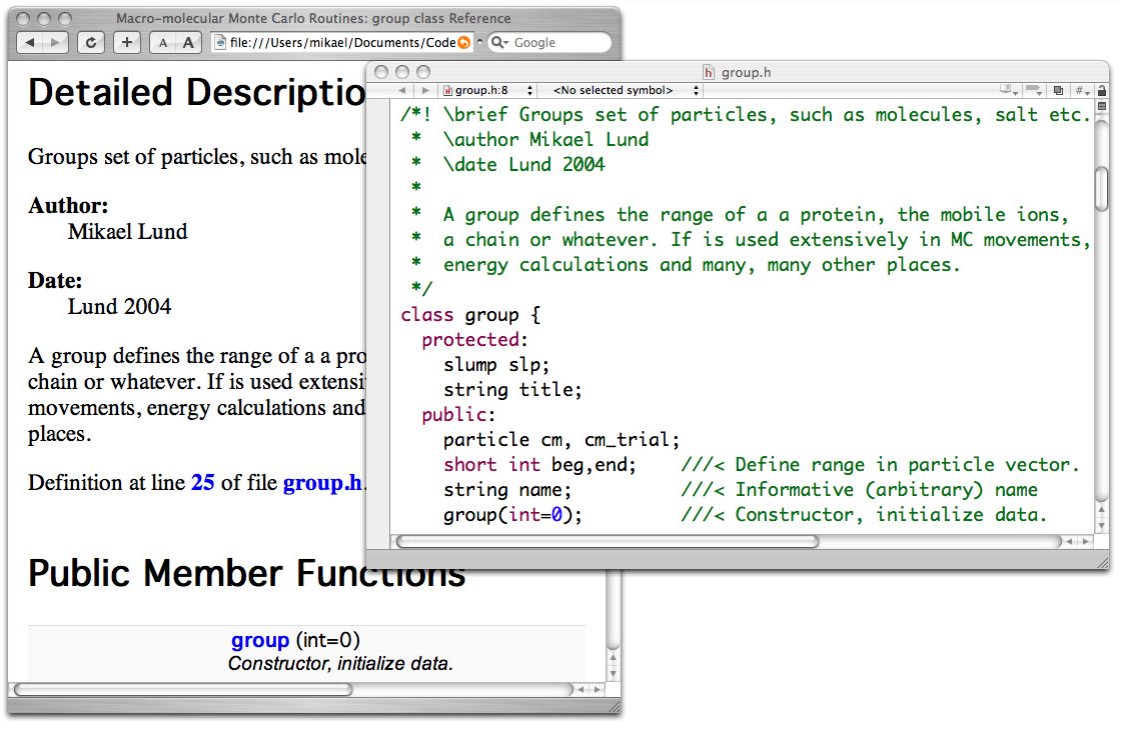
**Source code manual**. Code documentation through code comments. All code is commented with special keywords that are eventually collected into a web based manual.

We provide a class library and as such need to describe both what the classes do as well as how to use them. This can be conveniently achieved using a code documentation system – here we have chosen **Doxygen**[13] since (i) the documentation appears as normal code comments and (ii) the output is highly configurable, allowing LaTeX equations to be inserted etc. In Figure 2 we show how commented code is used to construct a web based manual of the available classes and functions in the code library. Another very useful feature of Doxygen is the ability to generate a graphical view of the class hierarchy. This enables the end programmer to visually see how a class is constructed as shown in Figure 1.

## Results and discussion

### General features

The class library provides simulation routines for ions, macromolecules and polymers in solution with a strong focus on electrostatic interactions using the primitive model of electrolytes where the solvent is treated as a structureless dielectric continuum [14]. It is, however, completely possible to expand the library to other systems, include explicit solvent etc. The routines have been developed over several years in connection with a number of scientific investigations, including proteins in solution [15]. As of writing, the code library contains general classes for the following,

• Explicit treatment of ions, including ion-ion correlation effects.

• Macromolecules – Proteins, flexible chains, charged surfaces.

• Charge regulation of molecules [16].

• Particle distribution functions and other statistical mechanical averages.

• Standard file formats are supported: PQR, Gromacs (GRO, XTC), Povray, XYZ.

An example of protein ionization will be presented later in the text. We stress that the project is under ongoing development and encourage interested users and developers to contribute.

#### "Trajectory" output

**Figure 3:**
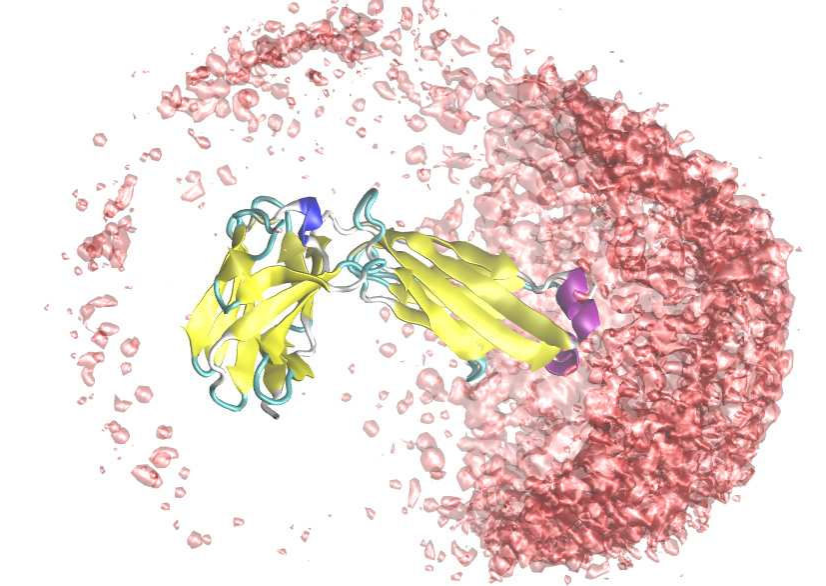
**Graphical analysis**. Lysozyme interacting with a fab-fragment – a simulation containing more than 340 amino acid residues as well as salt particles. The probability of finding lysozyme's mass-center around the the fab fragment is illustrated by the pink iso-surface. VMD [17] and Povray [20] was used to visualize the generated output from Faunus.

Molecular Dynamics simulation packages often save the time propagated particle trajectory to disk which is subsequently analyzed. In order to adopt this strategy we include an export routine that can write the simulated particle configurations to a compressed Gromacs XTC file [9]. This (sizable) file can be analyzed using the extensive set of tools provided in the Gromacs package, or visualized using VMD [17], for example. As an example of the latter, we have simulated lysozyme interacting with a fab-H fragment and, using VMD, plotted the spatial mass center distribution as shown in Figure 3.

### An example: Proton titration

**Figure 4:**
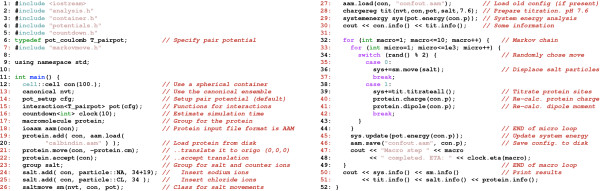
**Source code example**. Example of a Monte Carlo simulation program to calculate protein ionization states in an aqueous salt solution using explicit ions and the detailed three-dimensional protein structure.

Figure 4 shows – in 50 lines of code – a complete MC program for simulating the protonation state of a protein in a salt solution at a given pH value. Experimentally this corresponds to a standard potentiometric titration experiment where the net-charge is measured as a function of pH [18]. We will not go through all the lines in the code as the comments should be more or less self-explanatory. The overall program structure is

1. Set up the simulation cell (line 12)

2. Add protein(s) and ions (line 17–25)

3. Main loop with salt- and proton moves (line 32–49)

4. Print results and (line 50)

Results and comparisons with experimental data for such calculations can be found in a recent article [19]. The particles in the systems are clustered into groups and derived classes; there is a general group class (line 23) and a class for macromolecules (line 17). Note that we have also incorporated a general polymorphic class for markov moves and data analysis so that all derived classes have a common interface. For example, both the salt move class (line 36) and titration class (line 39) will store information about energy changes, if the move was a success etc. Data and analysis about each type of move is automatically shown by calling the respective information functions (line 51).

The source code for this and other examples are included with the class library and should serve as good starting points for developing new programs.

## Conclusion

A general class library for (macro-)molecular simulation is presented. We focus on Monte Carlo methods and the primitive model of electrolytes, although we see no technical limitations in expanding the project to cover other methods and molecular levels. The software design is object oriented, meaning that the code is extensively re-cycled which has several advantages:

• Programs can be developed in a modular manner (à la "Lego" bricks).

• Development and debugging is reduced.

• Memory requirements are minimized.

The class library is documented through extensive use of inline code comments. These comments are subsequently collected by a third party program (Doxygen) that will automatically construct a code manual and, hence, obsolete a separately maintained instruction book. In 50 lines of C++ code we demonstrate how to construct a complete MC program that can simulate protein protonation states in an aqueous salt solution. High performance in inner loops is established using Expression Templates, completely compatible with the flexibility and intuitive appeal of an object oriented design.

## Availability and requirements

*Project name*: faunus

*Project home page*: 

*Operating systems*: MacOS X, Linux, "Cygwin"

*Programming language*: C++

*Other requirements*: C++, Doxygen (optional)

*License*: GNU GPL

*Restrictions to use by non-academics*: GNU GPL

The latest version can be downloaded using the versioning control system "subversion" (SVN). On most UNIX type operating systems this is done by invoking the following shell command,

$ svn checkout



svnroot/faunus/trunk faunus

Prospective developers are welcome to contact the authors for write access to the online code repository, currently hosted by Sourceforge, Inc.

## Authors' contributions

ML did the Faunus software design and wrote the paper. BP and MT contributed to the implementation. All authors have read and approved the final manuscript.
